# Eliciting real cravings with virtual food: Using immersive technologies to explore the effects of food stimuli in virtual reality

**DOI:** 10.3389/fpsyg.2023.956585

**Published:** 2023-04-17

**Authors:** Nikita Mae Harris, Robert W. Lindeman, Clara Shui Fern Bah, Daniel Gerhard, Simon Hoermann

**Affiliations:** ^1^HIT Lab NZ, University of Canterbury, Christchurch, New Zealand; ^2^School of Product Design, University of Canterbury, Christchurch, New Zealand; ^3^Research and Innovation, University of Canterbury, Christchurch, New Zealand; ^4^School of Mathematics and Statistics, University of Canterbury, Christchurch, New Zealand

**Keywords:** virtual world, human-food interaction, eating, food cues, food cravings, cookies

## Abstract

In this paper, we explore the current technical possibilities of eating in virtual reality (VR) and show how this could be used to influence eating behaviors. Cue-based exposure therapy is a well-known method used to treat eating disorders. There are several benefits to using VR in combination with cue-based therapy. However, before VR-based cue-exposure can be used for therapeutic purposes, the ability of the VR environment to elicit craving responses in participants must be assessed. This was the objective of the first part of the study, where we assessed whether our VR environment elicited food craving responses in participants. Results showed that our VR environment elicited food craving responses: Salivation Magnitude, Food Craving State and Urge to Eat was significantly different from the neutral baseline. In addition, results showed that food cravings measured through the salivation magnitude in response to the virtual condition were not significantly different from the real condition, thus showing that VR had a comparable effect on producing food cravings. The second part of the study was conducted to determine whether the addition of olfactory and interaction cues in VR increased the development of food cravings. The results of this part showed that adding synthetic olfactory cues, paired with visual cues, to our system, provided a significant further increase in food cravings. Our results demonstrate that the use of food cues in VR can increase the development of food cravings and that it is possible to provide a simple yet convincing eating experience in VR. Inevitably, food interaction in VR is still underexplored territory and further research is needed to improve utility and application in disciplines related to food and eating.

## 1. Introduction

Food and eating are integral parts of our everyday lives. In our modern society, these are no longer just elements of our survival but have also evolved into forms that may define and differentiate us culturally and socially (Mintz and Du Bois, 2002). Rapid changes in our technology have also affected our eating behaviors and our perceptions of food. For more than two decades, scientists have shown how our preferences and liking for food are very much influenced by our perception of flavor, which is further influenced by sensory stimulation. These findings have encouraged many researchers to take advantage of this knowledge and apply it to their own disciplines. One of these is the field of Human-Food Interaction (HFI), or human-computer interaction research on food, which has emerged as an area of interest in the last few years. Associating the use of digital technologies to food and what is loosely referred to as “food practices,” for example, production, growing, shopping, eating, cooking, and disposal, have become central topics. In addition, studying how we behave around food and how we develop our eating behaviors using digital technologies might prove to be beneficial in improving our relationship with food.

### 1.1. Eating and virtual reality (VR) technology

Recently, there has been increasing interest in the potential benefits and new applications that may be obtained by incorporating digital technologies into traditional eating and drinking experiences (Ranasinghe et al., 2019). The range of possibilities offered by this technology is also reflected by the fact that experiences can be placed within the so-called reality-virtuality continuum (Milgram et al., 1995). This continuum ranges from the real environment (no VR elements) to augmented reality (real environment with some VR elements), and augmented virtuality (virtual environment with some real elements) to the virtual reality (only synthetic VR elements).

VR technology can be used to simulate naturalistic environments by delivering complex multi-sensory cues (proximal and contextual) and immersive human-computer interaction (de Carvalho et al., 2010). A head-mounted display and tracking system responds to user movement by changing the displayed scene in real-time as if one were looking around a real environment. Augmented Reality (AR) is an interactive experience of a real-world environment where the objects that reside in the real world are “augmented” by computer-generated synthetic perceptual information. Augmentation can occur across multiple sensory modalities, including visual, auditory, haptic, somatosensory, and olfactory. In other words, AR is used to enhance real environments or situations and offer perceptually enriched experiences. Immersive technologies simulating real-life environments can be effective tools in consumer testing, providing results with a higher external validity than laboratory testing and be a valid alternative to consumer testing in real-life situations (Sinesio et al., 2019). This rapid growth of VR technology means that research insights acquired today can be readily adapted and utilized to enhance consumer experiences or to change eating behaviors (Chen et al., 2020).

#### 1.1.1. Presence in VR environments

The basis of VR’s effectiveness is that users can be immersed in virtual environments as if they were real, such that experiences and behaviors learned in VR transfer into real-world contexts (Blascovich et al., 2002). Presence, or the psychological experience of existing within the VR environment, is the conceptual mediator through which many VR applications are typically believed to achieve these successes (Slater and Wilbur, 1997). In other words, presence is the tendency of people to respond to virtually generated sensory data as if they were real (Sanchez-Vives and Slater, 2005).

Presence can further be described using the concepts of place illusion and plausibility (Slater, 2009). The term ‘place illusion’ is reserved for the type of presence that refers to the sense of ‘being there’. It is the strong illusion of being in a place despite the sure knowledge that you are not there. Since it is a qualia, there is no way to directly measure it. However, indirect assessments based on employing questionnaires, interviews, physiological indices and behavioral responses have been used, all of which in some way compare responses with those expected in real experiences (Slater, 2009).

While place illusion is about how the world is perceived, plausibility is about what is perceived. Plausibility is the illusion that what is apparently happening is really happening (even though one knows for sure that it is not) (Slater, 2009).

When designing effective VR scenes to study eating behavior, it is essential to create a sense of presence so that users suspend disbelief and believe they are actually present in the VR environment and respond to stimuli as they would in equivalent real-life situations (Price and Anderson, 2007; Oliver and Hollis, 2021). Regardless of its importance, there is no generally accepted measure of presence, although the use of questionnaires is currently the favored approach (Grassini and Laumann, 2020).

Thus far, various physiological markers have been suggested as markers of presence, including heart rate, heart rate variability, skin conductance, skin temperature, and EEG, although their functionality has not been clearly determined (Grassini and Laumann, 2020).

To maximize the usefulness of immersive VR to study the effects of eating environment on eating behavior, it is vital that a participant feels that they are actually present in the simulated eating environment (Oliver and Hollis, 2021).

### 1.2. VR in food behavior research

Hartmann and Siegrist (2019) identified four main areas of food behavior research in which VR has been applied: food shopping behavior, the influence of environmental cues on eating behavior, the sensory evaluation of food, and the treatment of eating disorders in controlled environments. Of interest to us in this paper are the new possibilities for the implementation of cue exposure techniques in the treatment of eating disorders (Pla-Sanjuanelo et al., 2019).

#### 1.2.1. Eating behavior and VR

Our current real-world environment is characterized by the omnipresence of food cues. The sight and smell of real foods, but also graphical depictions of appetizing foods, can guide our eating behavior, for example, by eliciting food craving and influencing food choice (Blechert et al., 2014). Food cues thus appear to be an important influence on both eating and body weight particularly in overweight and dieting individuals who are most concerned with food and weight (Polivy et al., 2008).

Therapies such as Cognitive Behavioural Therapy (CBT) and Cue-Exposure Therapy (CET) have been shown to alleviate unhelpful eating behaviors by helping patients to modify their eating habits and to be mindful of their food intake. Food cravings (FCs) are intense urges to consume specific, usually energy dense foods such as chocolates, cookies, cakes and ice cream, regardless of physical hunger (Hill, 2007; Gearhardt et al., 2014) and have been positively associated with body mass index (Gilhooly et al., 2007), as well as overeating (Fedoroff et al., 2003). A better understanding of FCs may promote improved weight loss interventions (Ledoux et al., 2013).

Although there are a few studies around VR and food with regards to eating disorders or behavior (Gorini et al., 2010; Ferrer-Garcia and Gutierrez-Maldonado, 2013; Ledoux et al., 2013; Gutiérrez-Maldonado et al., 2018), most of them have been focused on visual stimuli alone. Only a few studies (Narumi et al., 2011; Arnold, 2017; Li and Bailenson, 2017) extended the study to chemical senses (olfactory and gustatory), which actually play a larger role in our eating experience (Spence et al., 2017). In terms of appetite and food cravings, the role of taste and smell may influence our food enjoyment and eating behaviors (Pelchat, 1997; Schiffman and Graham, 2000).

#### 1.2.2. Food cravings (FCs)

Humans typically crave energy-dense foods: chocolate and other chocolate-containing foods are the most frequently craved foods, followed by other high-caloric sweet and savory foods (Meule, 2020). For most people, the mere sight or smell of warm chocolate chip cookies initiates a strong desire to eat (craving). Such craving is a form of food cue reactivity: a conditioned response to food that is frequently accompanied by increased salivation, physiological arousal and neural activity in regions such as the ventral striatum (*VS*) (Boswell and Kober, 2016).

Research has shown that people respond neuro-biologically to food in the same way they do to addictive substances like tobacco and alcohol (Weingarten and Elston, 1990). A better understanding on what leads to cravings can lead to more successful interventions (Ledoux et al., 2013). The study of FCs is challenged by difficulty replicating the natural environment in a laboratory. VR could be used to deliver naturalistic cues in a laboratory. The Ledoux study investigated whether food related cues delivered by VR could induce greater FCs than neutral VR cues, photographic food cues, or real food. Experimental procedures involved delivering neutral cues *via* VR and food related cues *via* VR, photographs, and real food in counterbalanced order while measuring subjective (self-report) and objective (salivation) FCs. FCs produced by VR were marginally greater than a neutral cue, not significantly different from picture cues, and significantly less than real food. Our study builds upon the previous work of Ledoux et al. (2013). Ensuring that VR can be used to reliably induce the intended effect, such as food cravings, is of utmost importance and was an objective of the current study.

### 1.3. Sensory perception while eating

Current research suggest that our perception of flavor is multi-sensory. Hence, our eating experiences and perceived food flavor, whether pleasant or not, has been shown to be affected by the stimulation of gustatory, olfactory as well as other sensory modalities (Prescott, 2015; Spence, 2017).

As humans are visually-dominant beings, we begin this encounter with flavor by feasting with our eyes. Thereby, the popular adage “you eat with your eyes” (Hurling and Shepherd, 2003; Delwiche, 2012; Spence et al., 2016). Several studies have been conducted to show that visual cues prompt flavor or satiation expectations for our food (Wadhera and Capaldi-Phillips, 2014; Spence, 2015) The presentation of a dish on a plate, the cutlery used (Wansink et al., 2005), or even the packaging (Spence, 2018) influence the appeal of a given food, our behavior towards the food and even how satiated the food can make us feel. Visual food cues constitute a primary sensory input that allows predictions about the edibility and palatability of a food object (Blechert et al., 2014).

Once food is eaten, the perception of flavor moves to our chemical senses. The complete perception of flavor occurs through the multimodal interactions of gustatory and olfactory cues. In fact, the flavor that we perceive is largely influenced by our noses while our tongue provides us with taste qualities: sweet, sour, bitter, salty and umami (glutamate) (Spence, 2017). Within our mouths, we can also experience trigeminal sensations that help us determine certain characteristics of the food, such as temperature and texture (Thomas-Danguin et al., 2016). Auditory cues have also been shown to affect the perception of flavor. Zampini and Spence (2016) demonstrated how the crackly noises of potato chips affects our perception of their freshness. Spence (2014) also showed that background noises may impair our ability to enjoy sweet and sour tastes.

Out of the five basic human senses, sight, hearing, and touch are the sensory inputs predominately simulated in virtual environments (Wedel et al., 2020). Several studies have explored the interactions of our different sensory modalities and how each of these influences our eating experiences along with the aid of our rapidly changing technology (Bruijnes et al., 2016; Spence et al., 2017; Velasco et al., 2018; Crofton et al., 2019; Ammann et al., 2020).

### 1.4. Objectives of the study

Our study was designed to test whether similar circumstances hold true in VR with virtual food as it does in the real-world. If we are able to show that olfactory and interaction cues do indeed have an effect on food cravings, then this might be a good indication that it is worthwhile to further study eating in VR. Before VR-based cue-exposure can be used for therapeutic purposes however, the ability of VR scenarios to elicit craving responses in participants must also be assessed. These were the objectives of the present study. We hypothesize that the effects of VR cue exposure would have comparable effects on salivation, urge to eat and craving, to those of the real-world counterpart. Furthermore, we hypothesize that the addition of synthetic olfactory cues would increase the effects of cue exposure in VR conditions. Lastly, we also hypothesize that adding a means of manually interacting with the virtual food would further increase effects.

## 2. Materials and methods

### 2.1. Participants

Participants were recruited through advertisements (posters and flyers) distributed at the University of Canterbury and were screened for eligibility prior to take part based on the inclusion criteria (aged more than 18 years, no known food allergies, or no known feeding or eating disorders). The study was approved by the University of Canterbury Human Ethics Committee, and all participants gave written informed consent before commencing the study. Participants were asked to abstain from any food or drink two hours prior to their session.

Participants completed a self-reported demographics questionnaire on age, gender, ethnic background, height and weight. After completion of the study, participants were compensated with a shopping voucher.

### 2.2. Study design: Development of food cravings in VR

**Figure 1:**
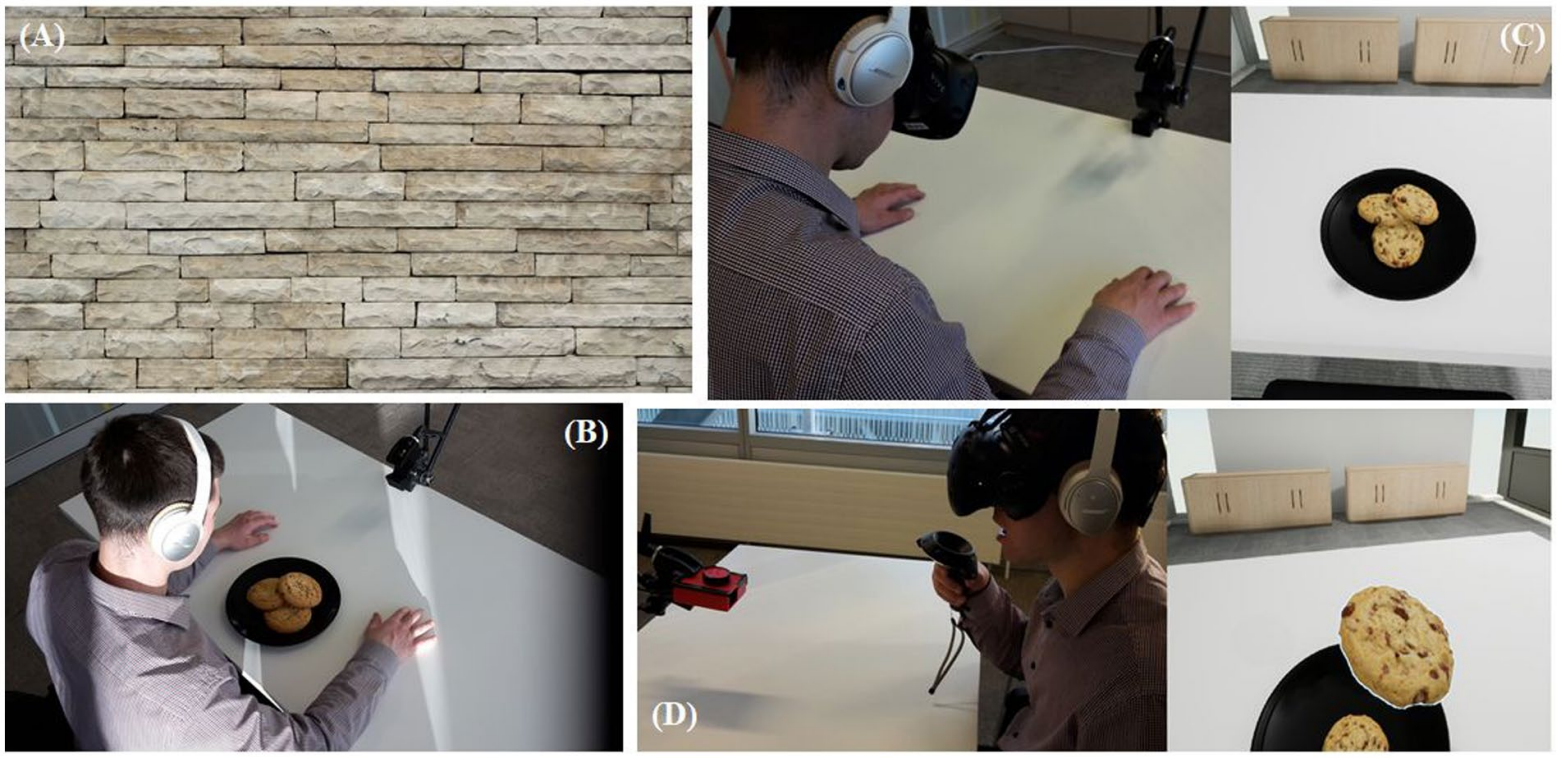
Experimental conditions and setup. **(A)** A user exposed to a white brick wall (NB condition). **(B)** A user exposed to real cookies (RC condition). **(C)** A user wearing head-mounted display and their view of the view of the virtual chocolate chip cookies in the virtual experiment room (VC condition). **(D)** A user exposed to virtual chocolate chip cookies with chocolate scent, holding a remote controller to interact with the virtual cookies (VCOI).

The design of this study was adapted from Ledoux et al. (2013). We used a within-subjects design and the order of the conditions was randomized using a Latin square. The neutral baseline was an exposure to an image of a static white brick wall (Figure 1A), whereas the other conditions all contained representations of chocolate chip cookies:

- Real chocolate chip cookies (RC)
- Virtual chocolate chip cookies (VC)
- Virtual chocolate chip cookies with chocolate scent (VCO)
- Virtual chocolate chip cookies with interaction (VCI)
- Virtual chocolate chip cookies with chocolate scent and interaction (VCOI)
- Neutral baseline (NB)

Our hypotheses are as follows:

- *H1a*: The exposure to Real Cookies (RC) evokes higher effects (i.e., urge to eat, salivation, craving) than to the Neutral Baseline (NB).
- *H1b*: The exposure to Virtual Cookies (VC) evokes higher effects (i.e., urge to eat, salivation, craving) than to the NB.
- *H2*: The effects of exposure to the VC are similar (statistically non-inferior) to the effects of RC.
- *H3a*: Adding a synthetic chocolate olfactory scent to the virtual cookie (VCO) has higher effects compared to VC alone.
- *H3b*: Adding six-degrees-of-freedom manual interaction possibilities to the virtual cookie (VCI) has higher effects compared to VC alone.
- *H4*: Virtual cookies with chocolate scent and interaction possibility (VCOI) evokes the highest effects compared to VC, VCO and VCI.

Our research adapted a three-arm experiment design often considered the gold standard for clinical trials of novel interventions. In our design, the exposure to the real cookies was chosen as the active control condition for which we, based on previous work, assumed efficacy to increase salivation, food cravings and urge to eat. Hence, H1a aims to validate our methods by replicating previous results. With H1b we analyzed whether our novel VR condition was also able to significantly increase craving responses.

In addition to H1 where difference of the VC compared to a neutral baseline was analyzed, in H2 we wanted to analyze if its effects were, within a range, and not worse than the RC active control condition.

With H3a and H3b, we explored whether the addition of more advanced possibilities with VR technology such as synthetic olfactory cues or the possibility to manually interact with the cookies further increased effects.

Finally, with H4 we wanted to explore whether adding olfactory cues and interaction possibilities at the same time led to the strongest effects in VR.

### 2.3. Experimental procedures

All participants were tested individually (see Figure 1 for overview of experiment setup). At the beginning of the experiment, participants were asked if they had abstained from any food or drink for two hours prior their session. This was done to ensure that prior fullness was not a confounding variable (Li and Bailenson, 2017). Once confirmed, participants were briefed about the experimental protocol and given the opportunity to ask questions. After that, they were asked to sign an informed consent form and complete a demographics questionnaire and a pre-task questionnaire. Among other questions, the pre-task questionnaire asked whether the participant had any experience with VR and whether they liked chocolate cookies, rated on a scale of 0 (not at all) to 100 (extremely).

Before completing each task, participants were asked to take three pre-weighed cotton dental rolls from a bowl and place two rolls buccally (between the cheek and the lower gums) and one sublingually (under the tongue) inside the mouth, targeting the parotid and sublingual salivary glands, respectively. They were also asked to wear the provided noise-canceling headphones to block out noise from outside.

In the conditions RC and NB participants had to sit down and observe the chocolate chip cookies (or a brick wall for NB). For the VR conditions which required interaction (VCI and VCOI), participants were provided with a head-mounted display (HMD, HTC Vive) and controllers that enabled them to pick up the virtual cookies. For conditions that included the use of the olfactory cues (VCO and VCOI), our scent device was only put in place after the participant had put on the VR headset (HTC Vive), so they were unaware of its presence. Moreover, following all conditions with olfactory cues, the researcher opened the windows and sprayed the room with an odor-neutralizer.

Each condition lasted for 2 min, after which participants were instructed to remove the cotton rolls from their mouth, put them on a provided dish, and have a sip of water as a palate cleanser between each task. After that, a two-minute break started, during which participants were asked to rate their urge to eat the cookies and to complete a food craving questionnaire. Meanwhile, the researcher weighed the used cotton rolls and recorded the measurements. The duration of the entire experiment varied between 30 and 45 min.

In this study, we measured food cravings responses with objective and subjective methods. Participants completed the Food Craving Questionnaire-State (FCQ-S) and rated their urge to eat cookies on a visual-analogue scale while we also used salivation magnitude as an objective measure.

### 2.4. Subjective measurements

The Food Craving Questionnaire-State (FCQ-S) was developed by Cepeda-Benito et al. (2000) and is a 15-item valid and reliable measure of state fluctuations in self-reported food cravings. Craving is defined as “an intense desire for a specific food that is difficult to resist” (Martin et al., 2008). The FCQ-S consists of five subscales including intense desire to eat, anticipation of positive reinforcement, anticipation of relief from negative states, lack of control over eating, and craving as a physiological state (Brockmeyer et al., 2015). Responses are scored on a Likert scale from 1 (strongly disagree) to 5 (strongly agree) and summed for a total FCQ-S score ranging from 15 to 75. High values indicate high state food cravings.

Participants also rated their Urge to eat Cookies (UC). The Urge to eat Cookies (UC) was measured using a Visual Analog Scale (Hill et al., 1991), which required participants to place a mark on a 100 mm line with one end (100 mm) indicating “extremely” intense craving and the other end (0 mm) representing “not at all.” Participants were instructed to make a mark at the point on the line that corresponded with their current craving experience for the cookie used in this study.

### 2.5. Objective measurements

The salivation magnitude (Epstein et al., 1995) was used as a physiological measure of food cravings. To physiologically measure food cravings, we used three cotton dental rolls (8 mm × 38 mm) to collect saliva produced by the participants. The salivation magnitude was derived by calculating the difference between the pre- and post-weights of the cotton dental rolls using precision scales.

### 2.6. Environment and equipment

**Figure 2:**
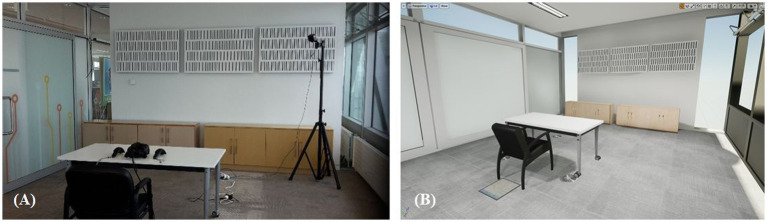
Real and virtual experimental rooms. **(A)** The actual experiment room. **(B)** Virtual replica of the experiment room rendered using Unreal Engine 4 (UE4).

Our virtual test environment was a virtual replica of our experiment room (Figure 2). We wanted to ensure that our participants would only concentrate on the task in front of them by making the environment quite uninteresting and as similar as possible to the room that they were physically in. The virtual room, table and plate were modelled using Maya 2017^1^ and then imported into Unreal Engine 4^2^, which was also used to render the graphics in the final virtual experience. In the virtual room, the objects that a participant would find were a table with a white surface and a black plate with three chocolate chip cookies on it. This environment was delivered using the HTC Vive, and its motion controllers were used to interact with the virtual cookies. We ran our experiment on an Intel Core i7-7700k 4.2GHz (eight cores) with 32GB of RAM and a NVIDIA GeForce GTX 1080Ti graphics card running Windows 10.

The real cookies used for the exposure were the Original Chocolate Chunk Cookies from Cookie Time Ltd. (Christchurch, NZ). These cookies were 85 g in weight and approximately nine centimeters in diameter. The virtual cookies were scaled to around the same size as the real ones. The black plate used was approximately 26.5 centimeters in diameter.

For the virtual cookie we used a high-polygon 3D model, with approximately 60,000 polygons, in real-world scale, purchased on turbosquid.^3^

**Figure 3:**
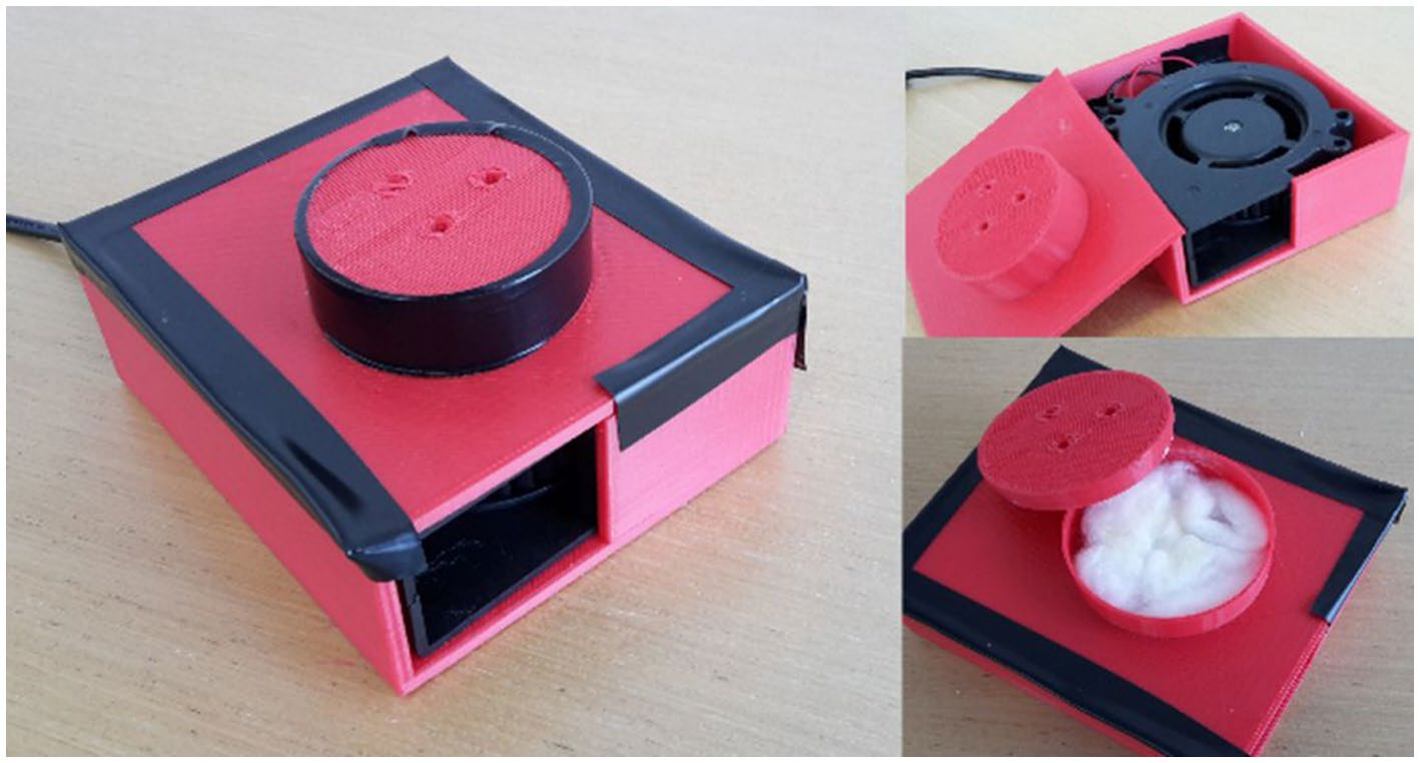
Olfactory device used to deliver the chocolate scent to the participant.

We also designed an olfactory device (Figure 3) to deliver the scent (Chocolate Fudge Fragrance Compound from CandleScience, Durham, USA) to the participant. This device comprised of a small blower fan which drew air from above, some cotton balls (approximately two shredded cotton balls weighing about 0.50 g each) soaked with chocolate scent oil (approximately 1 ml) and a red casing which was designed using Tinkercad and 3D printed in MakerBot Replicator 2. To neutralize the room from the chocolate scent, we used an odor-neutralizer (X-O Odor Neutralizer from Nixalite of America Inc., Illinois, USA).

### 2.7. Statistical analysis

SPSS 25 for Windows was used for data analysis. The alpha level for statistical significance was set to 0.05 and to 0.1 for marginal significance. Calculated salivation magnitude, FCQ-S and UC ratings were treated with a repeated measures ANOVA, testing within-subjects effects and pairwise comparisons. Sphericity was tested with Mauchly’s test and when violated, the degrees of freedom were corrected with Huynh-Feldt for *ε* > 0.75 and Greenhouse–Geisser for *ε* < 0.75. The effect of olfactory cues was tested using VC, VCO and VCOI, while the effect of interaction cues was tested using VC, VCI and VCOI with a repeated measures ANOVA.

## 3. Results

### 3.1. Participants’ characteristics

The study included a total of 30 participants (22 female, 8 male) from various ethnic background (15 identified as New Zealand European) with no known eating disorders (clinically diagnosed) or known food allergies. Eighteen participants were aged between 18 and 24 years, while 10 were between 25 and 34 years of age, and two were aged between 35 and 44 years. The average weight was 64.98 kg (SD = 15.35) with weights ranging from 45.10 to 104.6 kg. The average height was 164.7 cm (SD = 10.16). All participants stated that they do not smoke.

The average Body Mass Index (BMI) was 22.56 (SD = 2.91) for female and 26.85 (SD = 5.02) for male participants, which showed that one participant (female) was underweight, 20 (16 female) were of a healthy weight, seven (five female) were overweight, and two participants (both male) were considered obese, according to international BMI classification guidelines.

All participants were instructed to abstain from any food or drink for the 2 h prior to their scheduled session. All participants confirmed that they had not consumed any food 2 h prior to their experimental session, although 10 indicated that they had consumed a caffeinated beverage within 2 h prior to their session. One participant indicated having chewed gum recently and two participants indicated that they were currently on medication without specifying the details.

Most participants (19) stated that they generally liked chocolate chip cookies very much (>79 out of a 100), two participants reported a low liking (<39), whereas nine participants quantified their liking of chocolate chip cookies in the mid-range (40–79). Fourteen participants indicated that they had some prior experience with VR, whereas 16 participants stated that this was their first-time using VR. Nineteen participants stated that they generally liked chocolate chip cookies very much, two reported a low liking and nine quantified their liking of chocolate chip cookies in the mid-range.

### 3.2. Comparisons between VR and real-world exposure conditions

This section describes our results related to hypothesis H1 whether the effects of VR cue exposure have comparable results (food cravings) to those in real-world cue exposure. The VR condition described here is the virtual chocolate chip cookies (VC), while the real-world conditions were real chocolate chip cookies (RC), and the neutral baseline (NB).

#### 3.2.1. Salivation magnitude

The mean salivation magnitude measured for the real cookie (RC) exposure was 2.12 g (*S.D.* 1.19) compared to the neutral baseline (NB) at 1.72 g (*S.D.* 0.87). For the virtual cookie (VC) condition, a mean of 2.04 g (*S.D.* 1.41) was obtained (Table 1).

**Table 1:** Means and standard deviations (SD) of food craving measures for comparisons between VR and real-world exposure conditions.

	Salivation magnitude^1^	FCQ-S^2^	Urge to eat cookie^3^
	Mean ± SD	Mean ± SD	Mean ± SD
Real cookie	2.12 ± 1.19^a^	51.97 ± 14.33^a^	62.63 ± 27.76^a^
Virtual cookie	2.04 ± 1.41^a^	48.17 **±** 16.08^b^	42.03 ± 24.95^b^
Neutral baseline	1.72 + 0.87^b^	40.47 + 18.07^c^	20.37 + 24.16^c^

1 Salivation magnitude (measured in grams).

2 Food craving questionnaire state (scores from 15 to 75).

3 Urge to eat cookie (scores from 0 to 100).

Means that do not share a letter within a column are significantly different.

Mauchly’s test indicated that the assumption of sphericity had not been violated *X* ^2^(2) = 4.978*, p* = 0.083. Our results showed that there was significant difference between these exposure conditions, *F*(2, 58) = 3.185, *p* = 0.049, *η*^2^*_p_* = 0.099.

A pair-wise comparison showed that RC (*p* = 0.007) was significantly different from NB while VC was marginally significant (*p* = 0.061). There was no significant difference between RC and VC (*p* = 0.675, 95% CI = [−0.318, 0.484]).

#### 3.2.2. Food craving questionnaire-state

Out of a possible highest score of 75 and minimum score of 15, the mean FCQ-S score obtained for the condition using real cookies (RC) was 51.97 (*S.D.* 14.32), while the score for VC was 48.17 (*S.D.* 16.08) and the baseline condition NB obtained a mean score of 40.47 (*S.D.* 18.07), as shown in Table 1.

Mauchly’s test indicated the assumption of sphericity had been violated *X*^2^(2) = 10.671, *p* = 0.005, therefore degrees of freedom were corrected using Huynh-Feldt estimates of sphericity (*ε* = 0.793). Our analysis showed strong significant differences of FCQ-S scores between the exposure conditions, *F*(1.585, 45.969) = 17.03, *p* < 0.001, *η*^2^*_p_* = 0.370.

Pair-wise comparisons showed that the real cookie (RC) condition was significantly different from NB (*p* < 0.001) and VC (*p* < 0.001). There was a significant difference between RC and VC (*p* = 0.042, 95% CI = [0.146, 7.454]).

#### 3.2.3. Urge to eat cookies (UC)

Out of a possible highest score of 100, the mean UC score obtained for the condition using real cookies (RC) was 62.63 (*S.D*. 27.76), while the score for VC was 42.037 (*S.D.* 24.95) and the baseline condition NB obtained a mean score of 20.37 (*S.D.* 24.16), as shown in Table 1.

Mauchly’s test indicated that the assumption of sphericity had been violated *X*^2^(2) = 10.348, *p* = 0.006, therefore degrees of freedom were corrected using Huynh-Feldt estimates of sphericity (*ε* = 0.798). Our results showed participants felt significantly different levels of UC for the different conditions, *F*(1.596, 46.276) = 45.06, *p* < 0.001, *η*^2^*_p_* = 0.608.

Pair-wise comparisons showed that RC and VC were significantly different from NB (*p* < 0.001), There was a significant difference between RC and VC (*p* < 0.001, 95% CI = [13.935, 27.265]).

### 3.3. Effects of VR olfactory and interaction cues

This section describes our results related to our second and third hypotheses, where the addition of olfactory cues and interaction would increase the effects of VR cue exposure. The VR conditions included here are virtual cookies (VC), virtual cookies with scent (VCO), virtual cookies with interaction (VCI), and virtual cookies with scent and interaction (VCOI).

#### 3.3.1. Salivation magnitude

**Figure 4:**
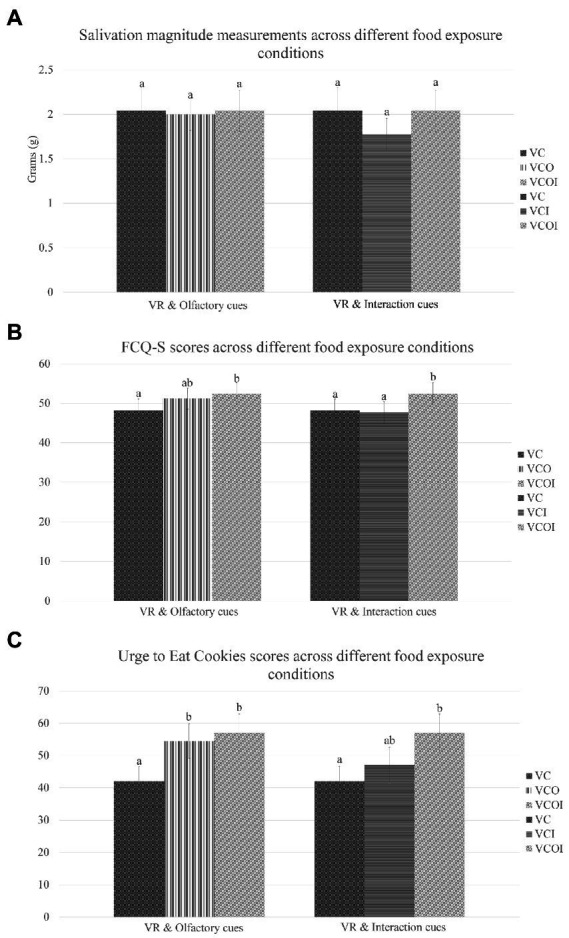
Means and standard errors of food craving measures comparing the effects of VR olfactory and interaction cues. **(A)** Results for Salivation Magnitude (measured in grams); **(B)** Results for FCQ-S - Food craving questionnaire state (scores from 15 to 75); **(C)** Results for UC – Urge to eat cookie (scores from 0 to 100). VC, Virtual Cookie; VCO, Virtual Cookie and Olfactory Cues; VCI, Virtual Cookie and Interaction; VCOI: Virtual Cookie, Olfactory Cues and Interaction. Means that do not share a letter are significantly different.

As mentioned above, the mean salivation magnitude obtained from virtual cookie exposure condition (VC) was 2.04 g (*S.D.* 1.41). In comparison, the mean salivation magnitude measured for the virtual cookie with scent (VCO) exposure was 2.00 g (*S.D.* 0.98), while the mean obtained for the virtual cookie with interaction (VCI) was 1.78 g (*S.D.* 0.99). For the virtual cookie with scent and interaction (VCOI) condition, a mean of 2.04 g (*S.D.* 1.27) was obtained (Figure 4A).

Neither the data from the three conditions compared to assess the effects of olfactory cues [*F*(2, 58) = 0.028, *p* = 0.973, *η*^2^*_p_* = 0.001] nor the three conditions with interaction cues [*F*(2, 58) = 1.110, *p* = 0.336, *η*^2^*_p_* = 0.037] were significantly different from each other in terms of salivation magnitude.

#### 3.3.2. Food craving questionnaire-state

Out of a possible highest score of 75, the mean FCQ-S score obtained for the condition using virtual cookies (VC) was 48.17 (*S.D.* 16.08), while the score for VCO was 51.2 (*S.D.* 14.53), VCI 47.67 (*S.D.* 15.61) and the VCOI condition obtained a mean score of 52.47 (*S.D.* 15.14), as shown in Figure 4B.

Food cravings were significantly different for the conditions that were compared with each other to assess the effect of olfactory cues, *F*(2, 58) = 4.478, *p* = 0.016, η^2^*_p_* = 0.134. In terms of olfactory cues, pairwise comparisons showed that VC was significantly different from VCOI (*p* = 0.047), while VC and VCO were not significantly different (*p* = 0.182). VCO and VCOI had no significant difference (*p* = 0.840).

Similarly, the conditions compared to analyze the effects of interaction cues were also significantly different, *F*(2, 58) = 6.226, *p* = 0.040, *η*^2^*_p_* = 0.177. VCOI showed significant differences from VC (*p* = 0.047) and VCI (*p* = 0.006), while VCI showed no significant difference from VC (*p* = 1.000).

This suggests that the addition of only interaction possibilities to the VR experience did not increase food craving (*p* = 1.000) and neither did the addition of only olfactory cues show clear increases (*p* = 0.182). However, the combined addition of interaction and olfactory cues increased food cravings significantly (*p* = 0.047).

#### 3.3.3. Urge to eat cookies (UC)

Out of a possible highest score of 100, the mean UC score obtained for the condition using virtual cookies (VC) was 42.03 (*S.D.* 24.95), while the score for VCO was 54.47 (*S.D.* 29.17), VCI 47.07 (*S.D.* 30.46) and the VCOI condition obtained a mean score of 57.07 (*S.D.* 31.50), as shown in Figure 4C.

UC ratings were significantly different for olfactory cues, *F*(2, 58) = 14.78, *p* < 0.001, *η*^2^*_p_* = 0.338. Pairwise comparisons showed that VCO (*p* < 0.001) and VCOI (*p* < 0.001) were significantly different from VC, while VCO and VCOI had no significant difference (*p* = 0.856).

Results showed that UC ratings were also significantly different for interaction cues, *F*(2, 58) = 10.27, *p* < 0.001, *η*^2^_p_ = 0.262. VC was not significantly different from VCI (*p* = 0.164) but had significant difference from VCOI (*p* < 0.001), while VCI and VCOI showed a trend towards a significant difference (*p* = 0.061).

This suggest that the addition of olfactory cues to the virtual experience increased the urge to eat cookies significantly (*p* < 0.001). However, the addition of only interaction possibilities did not significantly increase the urge (*p* = 0.164). The addition of both interaction and olfactory cues had the highest overall score, although the increase from just olfactory cues was not significant (*p* = 0.856).

#### 3.3.4. Summary of open-ended comments

Qualitative feedback data was summarized using thematic analysis (Nowell et al., 2017). The themes identified were “Bizarre,” “Like a game,” “Disappointing” and “Stimulating.” Some participants who had no prior experience with VR thought the setup was strange, but in a surprising way. One participant described the experience as “trippy.” Nevertheless, many of them thought the interaction was intuitive. Participants who had experience with VR and who also played games thought the interaction was more like a game. Most of them threw the virtual cookies across the room or just simply played with them. However, several participants stopped interacting with the virtual cookies after a few seconds. When asked for a reason, they described a feeling of disappointment at the knowledge that they were unable to eat the virtual cookies. Furthermore, a number of participants mentioned that in the neutral baseline conditions, they thought of the previous cookies that they were exposed to, which might explain why they still had an increased saliva production for this particular task. Overall, participants thought the chocolate scent was pleasant and some of them even mentioned it smelling like “freshly baked cookies.”

## 4. Discussion, limitations and future applications

Our study was adapted from a previous VR-cue exposure therapy (CET) study by Ledoux et al. (2013). In their study, when the baseline (a photo of nature) was delivered first, the salivation magnitude (SM) produced was significantly higher than the RC, food image, and VC exposures. In our study where the baseline was a white brick wall which was randomly ordered in the condition sequence, a difference in SM was found when RC and VC were compared to the baseline.

Prior to our study, we did a short pilot test to see how “neutral” we could make our baseline (data not shown). We initially tried photos of nature such as blooming flowers and mountains which made our pilot participants salivate more just like in the Ledoux study, therefore we decided to use a picture of a brick wall. Ledoux et al. (2013) theorized that in their study, because the neutral baseline cue exposure task was delivered first, the high salivation magnitude was likely due to residual saliva in the mouth rather than heightened reactivity to the nature scene. However, in our pilot study the conditions were randomized, therefore we do not know how to explain the phenomena of nature causing an increase in salivation, but we speculate that it could perhaps be a response based on human instincts. People perceiving nature may get a sense of “life” and when there is life, there is food, much like a flower may attract bees which we may correlate with honey. To the best of our knowledge, there is no study yet that has investigated such effects of nature on salivation.

Salivating is a physiological process which occurs mostly non-consciously (Winsor, 1930). It is controlled by the autonomic nervous system, which is stimulated while eating (Spence, 2011). While salivation naturally increases while eating to aid in the digestive process, it has also been shown to increase when one is exposed to the sights and smells of food cues, as a preparatory response (Spence, 2011). In the first part of this study, salivation appeared to change in response to both food cues (real or virtual). There was no significant difference between RC or VC. Evidence has suggested that salivation can be a useful physiological measure of consumer response to food cues because salivation is a signal of desire not under the consumer’s conscious control (Keesman et al., 2016). The benefit of measuring FCs with salivation is that it is a quantitative measure which is fairly easy and inexpensive to collect.

Although food cravings measured through FCQ-S in response to the virtual cookie (VC) condition were significantly lower from the real cookie (RC), they were significantly higher than the neutral baseline (NB). Similarly, in the urge to eat cookies measurement, the VC produced greater scores than the NB, but the real cookie produced a greater subjective UC than the VC, which could be anticipated. In summary this showed that both the real cookie and their VR counterpart were both effective in eliciting increased responses and thus supporting hypothesis 1. Hypothesis 2 was not fully supported since food craving and urge scores were significantly smaller and only the elicited salivation was of a similar magnitude.

In this study, real food cues produced more robust FCQ-S and UC responses than the virtual cookie; however, higher quality VR images, personalized VR environments, and incorporation of further multisensory experiences such as auditory and haptic cues may enhance the realism of the VR experience. This could then reduce the difference in effect to real food. While the complete replacement of real food with VR is still challenging, the use of real food also requires food storage, preparation facilities and can be costly given the perishable nature of real food (Ledoux et al., 2013). The benefit of using VR is that it offers fewer restrictions in use and has greater potential in simultaneously delivering food-related contextual cues compared to food pictures or real food.

Images of food are a standard laboratory method used to induce FCs for study, especially when brain imaging techniques are used to measure FCs (Polivy et al., 2008; Grimm et al., 2012). However, food pictures, much like real food, do not allow the study of the effect of food-related contextual factors on FCs (Ledoux et al., 2013). There have been varying results on the effect of VR food cues compared to food pictures. Gorini et al. (2010) studied the emotional responses of participants with eating disorders towards real food, VR food and photographs of food (PH). They found that when virtual food environments were viewed through an HMD, VR was as effective as real food, and more effective than the photographs in eliciting emotional responses. The authors argue that immersion and interaction are the key distinctive factors that make the difference between the VR and the PH conditions. In the PH condition, subjects are only able to passively observe static pictures, while in the VR condition they are able to interact by actively exploring the virtual environment, approaching the food and touching it virtually, thus prompting a stronger immersive response (Gorini et al., 2010). This would be one of the benefits of using VR over static food images.

In the second part of the study, we hypothesized that not only would olfactory cues influence the urge to eat in real life, but also in the virtual world. We envisioned that a VR system equipped with olfactory features would enhance the realism of the VR experience. We also considered that adding a means of interacting with the virtual food would further increase the development of food cravings. The results we obtained show that food cravings measured through salivation in response to olfactory and interaction cues were not significantly different from the baseline virtual cookie condition, while the subjective measures using FCQ-S and UC showed otherwise. If we disregard the salivation magnitude results and make VC the baseline condition for the VR exposure conditions, FCQ-S and UC ratings showed that olfactory and interaction cues were partially significant predictors of food cravings. This increase in food cravings from the baseline seemed to be more associated or stronger with olfactory cues than with interaction. The combination of both olfactory and interaction cues increased this further. This seems to indicate that olfactory cues may be enough of an addition to increase the development of food cravings. This finding is supported by other studies such as Fedoroff et al. (2003) who found that the smell of cookies baking increased cookie craving and cookie consumption for all participants. Olfactory and interaction cues in VR environments in relation to the consumption of donuts were studied (Li and Bailenson, 2017), and results showed that participants in the touch and scent conditions reported higher satiation compared to their counterparts. Existing studies on the subject have shown olfaction to be influential in VR experience as well. Most notably, scent stimuli have been shown to increase participants’ sense of presence in many, but not all, VR environments (Persky and Dolwick, 2020).

The differences between physiological and self-report outcome measures were of interest, especially as only the self-reported measures appeared to be altered by the introduction of interactive components. This could suggest that these measures assessed different aspects of food craving.

The modest effects seen in the current study may have been due to limitations in the VR system used. In this study, participants used controllers to pick up the cookies in the interaction conditions. Perhaps if participants saw their (virtual) hands picking up the (virtual) cookies, this could add to their sense of presence within the VR environment and the realism of the experiment. The sense of touch can also be a compelling factor in a virtual experience (Li and Bailenson, 2017). Hoffman et al. (1998) examined the influence of tactile feedback in virtual environments on how realistic participants perceived the experience to be. Participants who grabbed a real-world plate in their hands but saw a virtual representation of it through a head-mounted display (HMD) perceived the plate to be heavier and more likely to obey gravity than those who saw the plate only in VR. In our feedback summary, several participants stated that they stopped interacting with the virtual cookies after a few seconds. A more tactile way of interaction such as touching the actual plate and cookies but seeing it in an immersive environment could be studied to see if it leads to a longer interaction. Olfaction is not routinely integrated into VR environments; however, dedicated hardware tools are emerging to make this possible. It is important to conduct research to understand under which specific circumstances olfactory stimuli are likely to add to user experience and help achieve the goals of a VR application (Persky and Dolwick, 2020).

The open-ended comments made by the participants indicated that they were able to interact with the VR system and should be an important consideration for future research as often the goal of using VR is to make the experiment more engaging and ecologically valid. In addition, assessing and measuring participants’ presence and comfort in the virtual environment could also be related to the food craving results. Presence can be defined as the psychological state in which virtual objects are experienced as actual objects in either sensory or non-sensory ways (Lee, 2004). Chen et al. (2020) reflected that it was possible that the VR environments presented in their study were not comfortable enough to increase food craving, as the average comfort rating for their environments were either “slightly comfortable” or “moderately comfortable.”

A further limitation to this study is that self-reported measures may be influenced by factors such as human error, bias, or intentional misrepresentation. For these reasons, objective measures of FCs are desired. While the objective salivation magnitude measurements used in this study did not show any clear differences between food exposure conditions, future research could explore the use of VR in studying FCs together with brain imaging techniques. Lastly, another limitation of this study may be its small sample size.

## 5. Conclusion

With these findings, we conclude that the addition of olfactory cues, as well as the combination of olfactory and interaction cues, can affect food cravings in VR and can influence our VR eating experiences. The effects of interaction cues on its own in a VR setting, with regards to food craving, should be explored further. Future research should also continue to develop and test more sophisticated VR programs and environments. Another aspect that can be considered in future experimental VR set-ups are multisensory experiences that combine various other stimuli. This paper did not include auditory, gustatory such as tasting the cookie, or tactile feedback such as touching and chewing the cookie or proprioceptive stimuli. Exposing participants to cross-modal multisensory stimuli may result in an even more realistic experience and higher levels of presence, which could lead to more significant results in VR.

The results of our study show that the increase in the development of food cravings is a positive indication that we can have similar eating experiences in VR to those of the real world. The field of HFI, particularly in the VR setting, is relatively young. Definite design practices must be set within the community to make this research exploration effective. When it comes to food and dining, there are a lot of challenges that we still must overcome before we can create compelling eating experiences in the virtual world. VR offers an infinite array of sensory food stimuli and allows for the manipulation of a product’s features without affecting environmental features.

## Data availability statement

The anonymized data supporting the conclusions of this article will be made available from the corresponding author SH upon reasonable request.

## Ethics statement

The research involving human participants was reviewed and approved by University of Canterbury Human Ethics Committee. The participants provided their written informed consent to participate in this study. Written informed consent was obtained from the individual(s) for the publication of any potentially identifiable images or data included in this article.

## Author contributions

NH, SH, and RL: conceptualization. NH and SH: methodology. NH: investigation, data curation, and writing – original content. SH and CB: data analysis. CB, SH, and RL: drafting and editing manuscript. DG: data analysis advisor. SH and RL: supervision. RL: funding acquisition. All authors have read and agreed to the final version of the manuscript.

## Funding

This research was funded by the HIT Lab NZ, University of Canterbury, New Zealand.

## Conflict of interest

The authors declare that the research was conducted in the absence of any commercial or financial relationships that could be construed as a potential conflict of interest.

## Publisher’s note

All claims expressed in this article are solely those of the authors and do not necessarily represent those of their affiliated organizations, or those of the publisher, the editors and the reviewers. Any product that may be evaluated in this article, or claim that may be made by its manufacturer, is not guaranteed or endorsed by the publisher.

## Footnotes

^1^ www.autodesk.com

^2^ www.unrealengine.com

^3^ www.turbosquid.com
